# Supplementary material for: Evidence supported by Mendelian randomization: impact on inflammatory factors in knee osteoarthritis
Source: Front Med (Lausanne). 2024 May 28;11:1382836. doi: 10.3389/fmed.2024.1382836 (PMC11165061; doi:10.3389/fmed.2024.1382836)
Supplement: Supplementary file 1 [file Data_Sheet_1.ZIP › data/GCST90274842_5e6/842.forest.pdf]

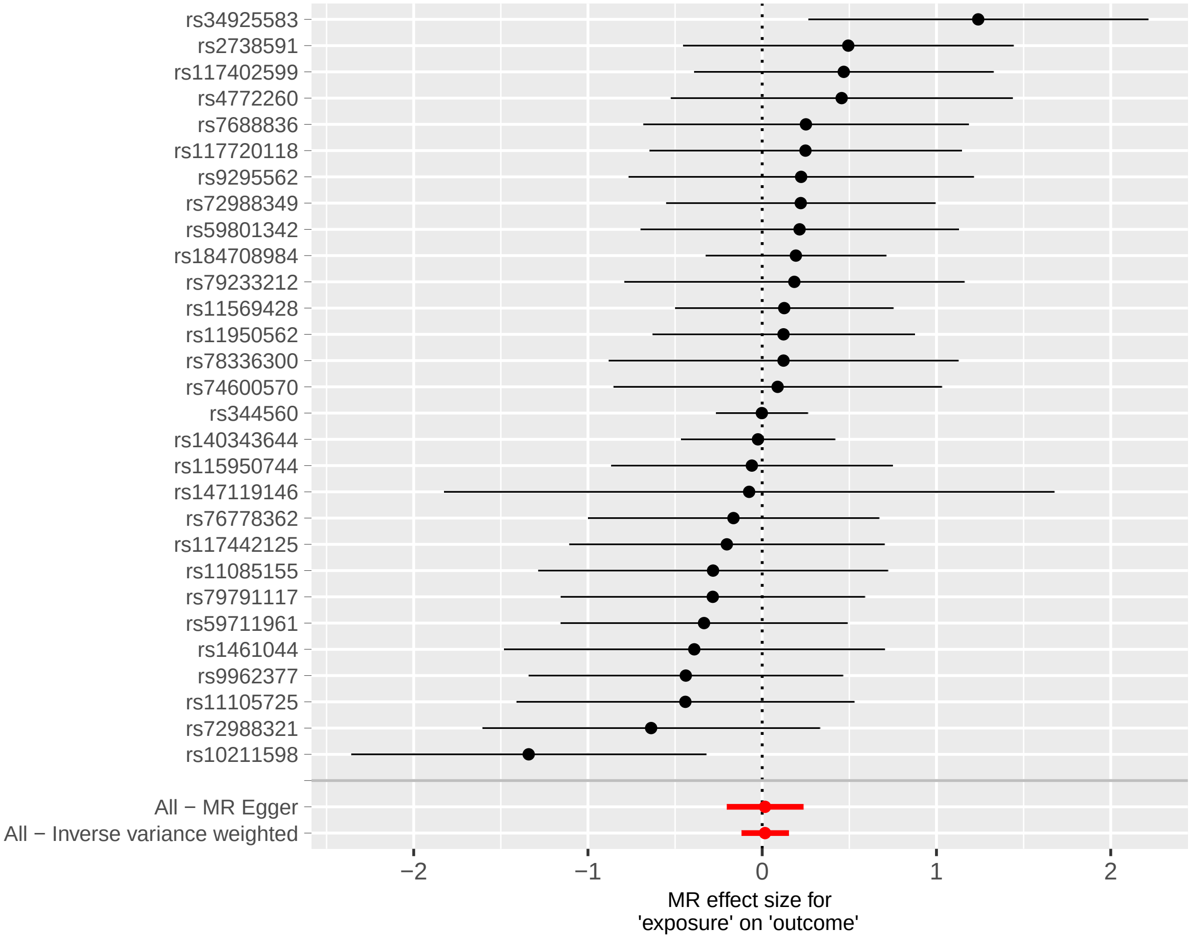
rs34925583
rs2738591
rs117402599
rs4772260
rs7688836
rs117720118
rs9295562
rs72988349
rs59801342
rs184708984
rs79233212
rs11569428
rs11950562
rs78336300
rs74600570
rs344560
rs140343644
rs115950744
rs147119146
rs76778362
rs117442125
rs11085155
rs79791117
rs59711961
rs1461044
rs9962377
rs11105725
rs72988321
rs10211598
All − MR Egger
All − Inverse variance weighted
−2
−1
0
1
2
MR effect size for
'exposure' on 'outcome'
